# STRN-ALK Fusion in Lung Adenocarcinoma with Brain Metastasis Responded Well to Ensartinib: A Case Report

**DOI:** 10.3390/curroncol29100530

**Published:** 2022-09-21

**Authors:** Linlin Zhang, Ping Xiao, Fanlu Meng, Diansheng Zhong

**Affiliations:** Department of Medical Oncology, Tianjin Medical University General Hospital, Tianjin 300052, China

**Keywords:** STRN-ALK fusion, lung adenocarcinoma, brain metastasis, ensartinib

## Abstract

STRN-ALK fusion is a rare ALK rearrangement identified in non-small cell lung cancer (NSCLC) patients. Here, we reported a case of lung adenocarcinomas with brain metastasis, harboring STRN-ALK fusion, responded well to ensartinib. This case report could provide more information for the therapeutic strategy selecting of NSCLC patients harboring STRN-ALK fusion.

## 1. Introduction

EML4-ALK fusion is the most common ALK rearrangement detected in non-small cell lung cancer (NSCLC) patients. Effectiveness of ALK tyrosine kinase inhibitors (TKIs) (crizotinib, alectinib, brigatinib, et al.) in NSCLC patients with EML4-ALK fusion could be easily obtained from randomized controlled trials. However, response of NSCLC with non-classic ALK fusions to ALK-TKIs has rarely been reported. 

Ensartinib is a second-generation small molecule inhibitor that inhibits ALK tyrosine kinase activity. eXalt3 trial demonstrated ensartinib had superior efficacy as compared with crizotinib for the treatment of ALK-positive NSCLC patients [1]. In addition, preclinical investigation showed that many rare ALK fusion variants exhibited good response to ensartinib [2]. Here, we described a case of NSCLC with brain metastasis harboring STRN-ALK fusion responded well to ensartinib. The aim of this report is to provide more evidence for therapeutic strategy selecting for the treatment of NSCLC patient due to STRN-ALK fusion.

## 2. Case Report

**Figure 1:**
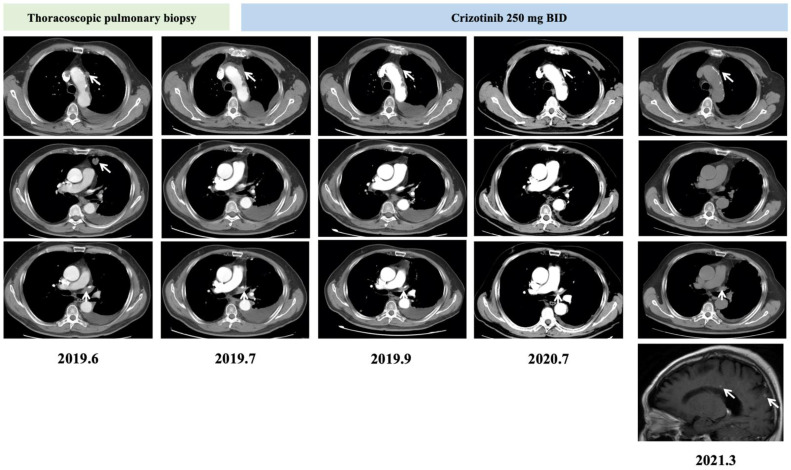
Thoracic computed tomography (CT) and brain MRI images recorded treatment-related changes of the patient under crizotinib.

A-67-year old male patient presented with chief complaint of cough for 1 month in June 2019. He had a history of smoking for 40 years and no alcohol consumption. Computed tomography (CT) demonstrated: (1) a nodule of 20 × 19 mm in his upper lobe of the left lung; (2) nodular thickening of both side of the pleura; (3) mediastinal lymph nodes enlargement; (4) left pleural effusion (Figure 1). No bone, liver, brain and other metastasis were detected. Tumor biomarker carcinoembryonic antigen (CEA) was at high level of 38.84 ng/mL. Thoracoscopic pulmonary biopsy was conducted, and the nodule in left upper lobe of the lung was removed for pathological analysis. Pathology results suggested lung adenocarcinoma. Thus, the diagnosis of the patients was stage IVa (cT1aN2M1a) lung adenocarcinoma. 

**Figure 2:**
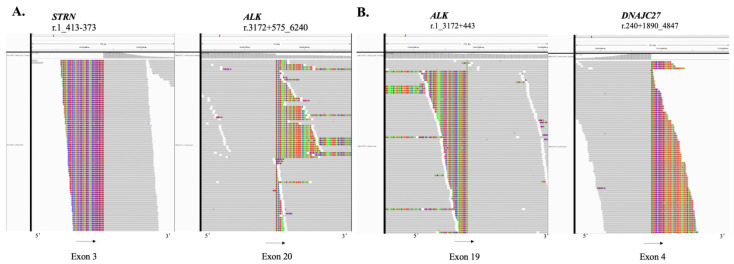
(**A**) DNA next generation sequencing showed the fusion of exon 3 of STRN with exon 20 of ALK. (**B**) DNA next generation sequencing showed the fusion of exon 19 of ALK with exon 4 of DNAJC27.

According to NSCLC guidelines, molecular testing was preformed using patient’s tissue sample. DNA next generation sequencing (NGS), including 825 cancer-related genes panel, revealed an STRN:exon3-ALK:exon20 fusion with variant allele frequency (VAF) 24% (Figure 2A), an ALK:exon 19-DNAJC27:exon 4 fusion with VAF 9.1% (Figure 2B), and other potential cancer related mutations (Table 1). Tumor mutation burden was 2.38 mutations/Mb. Immunohistochemistry showed negative expression of programmed death-ligand 1 (PD-L1). Raw data generated from targeted sequencing can be obtained from CNCB-NGDC (http://bigd.big.ac.cn/gsub/ accessed on 1 August 2022) with accession number HRA002593.

Crizotinib was administrated as first-line treatment of this patient, with a dose of 250 mg twice daily, according to previous case reports at that time. The patient responded well two months post-treatment (Figure 1). And meanwhile, serum CEA level decreased to 8.25 ng/mL. Eighteen months later, asymptomatic brain metastasis was detected by MRI examination (Figure 1). At the same time, the serum CEA level also increased to 41.19 ng/mL. 

**Figure 3:**
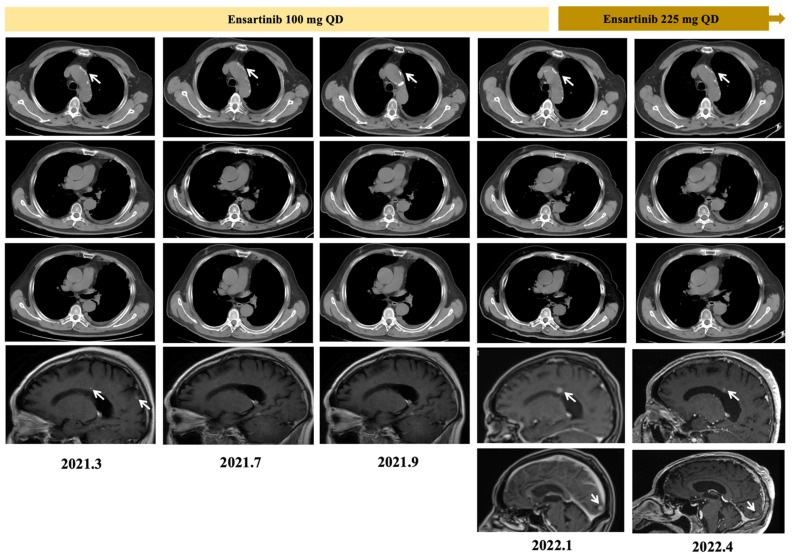
Thoracic computed tomography (CT) and brain MRI images recorded treatment-related changes of the patient under ensartinib.

Simultaneously, DNA-NGS assay of patient’s cerebrospinal fluid biopsy was performed, but no resistance mutations was identified. Ensartinib was administrated as second-line treatment, with a dose of 225 mg once daily. However, dose reduction was conducted because of persistent drug-induced fever (after differential diagnosis with infection and other reasons), to 100 mg once daily. Two months after switch to ensartinib, MRI showed his brain metastasis disappeared, and serum CEA level reduced to 9.60 ng/mL (Figure 3). This response lasted for ten months when his brain metastasis progressed again; but mediastinal lymph nodes kept stable, and the serum CEA level was still at 9.60 ng/mL. Surprisingly, when the dose of ensartinib increased to 225 mg once daily, the patient’s brain metastasis responded again, without drug-related fever (Figure 3). 

## 3. Discussion

STRN-ALK fusion is a rare type of ALK rearrangement, especially in NSCLC. Treatment design for NSCLC patients with rare ALK fusion usually depended on previous case reports. In recent years, several cases of NSCLC with STRN-ALK fusion have been reported (Supplementary Table S1). From these literatures, we found most of the NSCLC patients harboring STRN-ALK fusion were male (8/9), non-smoker (7/9) and adenocarcinoma (9/9) [3,4,5,6,7,8,9,10,11]. All the STRN-ALK transcript consisted of the fusion between exon 3 of STRN and exon 20 in ALK [3,4,5,6,7,8,9,10,11]. STRN-ALK fusion presented as a driver alteration in 7 of the 9 reported NSCLC patients [3,4,6,7,8,9,10]. Three of them were reported to receive crizotinib and response well to it [3,8,10]. However, the response to alectinib from different reports demonstrated inconsistent results [4,6,7,8,9,10]. In addition, STRN-ALK fusion was found to be co-existed with EGFR activating mutation and EGFR T790M mutation, in two osimertinib acquired resistant NSCLC patients. Crizotinib combined with EGFR-TKIs demonstrated good efficiency in these two patients [5,11]. Apart from alectinib and crizotinib, efficiency of other ALK-TKIs in NSCLC patients with STRN-ALK rearrangement has not been reported before.

Ensartinib is a second-generation ALK-TKI. In the phase I/II eXalt2 trial, ensartinib demonstrated to be associated with high systemic as well as central nervous system response rates in patients harboring ALK fusion who resistant to prior crizotinib treatment [12]. In addition, it is reported that ensartinib displayed lower IC50 in several rare partner-ALK fusions, but these fusions did not include STRN-ALK fusion [2]. In this case, we found ensartinib demonstrated good clinical efficiency in NSCLC patients with STRN-ALK fusion, especially brain metastasis, relapsed from crizotinib. In addition, the standard therapy of brain metastases nowadays remains stereotactic radiation, but in this case, we found targeted therapy could be an option for small asymptomatic lesions. Furthermore, there was another ALK: exon 19-DNAJC27: exon 4 fusion detected in this case. However, this fusion resulted in ALK gene tyrosine kinase region (ALK exon 20-29) lost, so that, it was not a target of any ALK-TKIs (Figure 2B).

In conclusion, this case provided a valuable clinical evidence of NSCLC patient with brain metastasis harboring STRN-ALK fusion treated effectively with ensartinib.

## Figures and Tables

**Table 1:** Other genetic alterations detected in the patient’s tumor sample through DNA-NGS assay.

Gene Name	Nucleotide Change	Amino Acid Change	Mutation Type	Variant Allele Frequency (%)
ALK	c.2920G > C	p.E974Q	SNV	42.7
EPHA5	c.188T > C	p.L63S	SNV	30.4
PRKDC	c.9587G > T	p.K3196N	SNV	20.3
PFWD2	c.268A > T	p.S90C	SNV	29.2
RPL5	c.262_264delinsTTTT	p.V88Ffs*2	Deletion	26.5

SNV: single nucleotide variation.

## Data Availability

Raw data generated from targeted sequencing can be obtained from CNCB-NGDC (http://bigd.big.ac.cn/gsub/ accessed on 1 August 2022) with accession number HRA002593.

## Supplementary Materials

The following supporting information can be downloaded at: https://www.mdpi.com/article/10.3390/curroncol29100530/s1, Table S1: Case reports of NSCLC patients harboring STRN-ALK fusion.
