# Supplementary material for: STRN-ALK Fusion in Lung Adenocarcinoma with Brain Metastasis Responded Well to Ensartinib: A Case Report
Source: Curr Oncol. 2022 Sep 21;29(10):6749–53. doi: 10.3390/curroncol29100530 (PMC9601241; doi:10.3390/curroncol29100530)
Supplement: Supplementary file 1 [file curroncol-29-00530-s001.zip › curroncol-1912088-supplementary.pdf]

Supplementary Table S1. Case reports of NSCLC patients harboring STRN-ALK fusion

Author,Publication Year	Gender	Age (Years)	Smoker	Pathology	Stage	STRN-ALK variants	Co-mutations	PD-L1 expression level	Treatments, Lines	Response	Duration of response (months)
Yan Yang et al, 2017 [3]	Male	59	Never	Adenocarcinoma	IV	S3:A20	MYC amplification<br>TP53 R181C	Not reported	Crizotinib, Third	CR	6, ongoing
Yuko Nakanishi et al, 2017 [4]	Male	51	Never	Adenocarcinoma	IV	S3:A20	--	Not reported	Alectinib, First	PD	--
Chunhua Zhou et al, 2019 [5]	Male	43	Smoker	Adenocarcinoma	IV	S3:A20	EGFR ex19del,<br>EGFR T790M	Not reported	Gefitinib+crizotinib, Third	PR	6
Cuiyun Su et al, 2020 [6]	Male	64	Never	Adenocarcinoma	IV	S3:A20	GRM8 E508K<br>SETD2 E1553K	Negative	Alectinib, First	PR	19, ongoing
Misako Nagasaka et al, 2020 [7]	Male	66	Never	Adenocarcinoma	IV	S3:A20	TP53 L43fs<br>MYC amplification	98% (22C3)	Alectinib (after 1 cycle of chemotherapy), First	CR	6, ongoing
Mengnan Li et al, 2021 [8]	Male	42	Never	Adenocarcinoma	IV	S3:A20	Met amplification<br>TP53 C991T<br>TP53 C742T<br>BRCA1 A5347C	Not reported	Alectinib, First<br>Crizotinib, Second	PR for alectinib<br>PR for crizotinib	4, for alectinib;<br>11, on going, for crizotinib
Hao Zeng et al, 2021 [9]	Female	29	Never	Adenocarcinoma	IV	S3:A20	PDK1-ALK (P7: A20)<br>TP53 ex9 splicing mutation	Not reported	Alectinib, First	PR	7, ongoing
Kunyan Sun et al, 2021 [10]	Male	65	Smoker	Adenocarcinoma	IV	S3:A20	PIK2CA G106V<br>KRAS G12C<br>TP53 R267H	Not reported	Alectinib, First<br>Crizotinib, Second	PD for alectinib<br>PR for crizotinib	--<br>4, for crizotinib
Qian Zeng et al, 2021 [11]	Male	38	Never	Adenocarcinoma	IV	S3:A20	EGFR L858R<br>EGFR T790M<br>RB1 R445<br>TP53 T284Afs62<br>EML4-ALK fusion	Not reported	Osimertinib+crizotinib, Third	SD	5, ongoing

CR: complete response; PR: partial response; SD: stable disease; PD: progressive disease
